# Carbon Capture Utilization for Biopolymer Foam Manufacture: Thermal, Mechanical and Acoustic Performance of PCL/PHBV CO_2_ Foams

**DOI:** 10.3390/polym13152559

**Published:** 2021-07-31

**Authors:** Kayode E. Oluwabunmi, Weihuan Zhao, Nandika Anne D’Souza

**Affiliations:** 1 Department of Mechanical and Energy Engineering, University of North Texas, Denton, TX 76207, USA; KayodeOluwabunmi@my.unt.edu (K.E.O.); Weihuan.Zhao@unt.edu (W.Z.); 2 Department of Materials Science and Engineering, University of North Texas, Denton, TX 76207, USA

**Keywords:** polyhydroxy-butyrate-*co*-valerate, polycaprolactone, insulation foams, sub-critical CO_2_, thermal conductivity modeling, sound absorption, mechanical properties

## Abstract

Biopolymer foams manufactured using CO_2_ enables a novel intersection for economic, environmental, and ecological impact but limited CO_2_ solubility remains a challenge. PHBV has low solubility in CO_2_ while PCL has high CO_2_ solubility. In this paper, PCL is used to blend into PBHV. Both unfoamed and foamed blends are examined. Foaming the binary blends at two depressurization stages with subcritical CO_2_ as the blowing agent, produced open-cell and closed-cell foams with varying cellular architecture at different PHBV concentrations. Differential Scanning Calorimetry results showed that PHBV had some solubility in PCL and foams developed a PCL rich, PHBV rich and mixed phase. Scanning Electron Microscopy and pcynometry established cell size and density which reflected benefits of PCL presence. Acoustic performance showed limited benefits from foaming but mechanical performance of foams showed a significant impact from PHBV presence in PCL. Thermal performance reflected that foams were affected by the blend thermal conductivity, but the impact was significantly higher in the foams than in the unfoamed blends. The results provide a pathway to multifunctional performance in foams of high performance biopolymers such as PBHV through harnessing the CO_2_ miscibility of PCL.

## 1. Introduction

Energy generation, consumption, and other industrial processes that burn fossil fuels such as oil, gas, and coal have been attributed to be largely responsible for the excess concentration of anthropogenic greenhouse gases, specifically atmospheric CO_2,_ contributing to global warming [1,2,3]. With current global emissions of CO_2_ expected to reach 44 gigatons per year from its current value of 27 gigatons, scientists believe that missing the Kyoto target might exacerbate the already worsening global warming trend. Sequestering CO_2_ brings with it many challenges in storage. There is a growing interest in developing a net zero approach for utilization of generated CO_2_ in products. This is driving the increased use of CO_2_ and other anthropogenic gas emissions from Carbon Capture and Storage (CCS) to Carbon Capture and Utilization (CCU) [4,5,6]. The Congress of the United States, through its bipartisan act of 2018, has earmarked potential pathways for the utilization of CO_2_ [7]. Using the Department of Energy (DOE) as its funding vehicle, four key areas of research have been identified for enhancing the utilization of CO_2_ gas for the development of various products. These are in the production of cement for building and construction, conversion of CO_2_ to carbonates through the process of mineralization, enhanced oil and gas recovery, and the production of polycarbonate plastics [8,9]. With foams being a billion-ton market, using CO_2_ as a manufacturing means is attractive. The pairing of biopolymers being manufactured into foams using CO_2_ provides a novel intersection of utilizing captured CO_2_ for value added products.

Foams and porous structures are fast becoming versatile for various applications [10,11,12]. They have been used extensively in sectors such as packaging [13], furniture and automotive parts [14,15,16], medical scaffolds and sutures [17,18,19], and in building and construction materials [20,21]. Polyurethane and styrofoam are the industry standard materials used in manufacturing microcellular foams. However, due to environmental issues caused by the disposal of these petroleum-based polymeric foams, researchers have intensified efforts in developing suitable eco-friendly foams from bio-resourced polymers as possible alternatives [22,23,24].

Poly 3-hydroxybutyrate-co-3-hydroxyvalerate (PHBV) is a semi crystalline co-polymer that has received considerable research interest in recent times [25]. It is mainly produced from the bacterial fermentation of energy reserves and carbon sources of various feed stocks, such as plant oils and sugars that have been cultured under unbalanced growth conditions [26,27]. PHBV was developed as an improvement on the homopolymer poly(3-hydroxybutyrate) (PHB). The industrial applications of PHB are limited due to its low impact strength, stiffness, and excessive brittleness. To improve on this, the monomer, hydroxy valeric acid, was introduced into its molecular structure through co-polymerization [28,29,30]. As a result of this, PHBV, a more flexible polymer with greater ductility and toughness, was developed [31,32]. PHBV’s biological compatibility and barrier to water, air, and aroma has made it a potential polymer with promising applications for disposable packages and medical uses [33]. However, its low melt viscosity, high crystallinity, and immiscibility when blended with other polymers still limits the ease of converting it into microcellular foams [23]. Various attempts have been made to improve the foaming properties of PHBV. It has been blended with different polymers and fillers to produce lightweight composites with a wide range of cellular morphologies using both physical and chemical foaming techniques [23,33]. For instance, Le Moigne et al. [34] developed a blend of PHBV with organo-clay foams using the extrusion foaming process with supercritical CO_2_ (sc-CO_2_) as the blowing agent. SEM imaging showed that the presence of clay led to a more homogenous morphology in the blend. Furthermore, an increase in clay concentration in the blend was also reported to have led to about 50% improvement of the foam porosity. A very narrow foaming window that favored homogenous nucleation of the pores was observed. Similarly, a more uniform morphology was observed when PLA of up to 25 wt.% was blended with PHBV and foamed with subcritical CO_2_ gas using the batch process [35]. Other researchers have also reported an open cell morphology when PHBV was foamed with sodium bicarbonate using the chemical foaming extrusion process [36,37]. PHBV has been reported to be a brittle semicrystalline polymer with limited mechanical applications, hence the need to blend it with other polymers and fillers. The degree of interfacial adhesion between fillers and the polymer matrix has been reported to be responsible for the mechanical properties displayed by PHBV foam blends. Peng et al. [33] recorded an improvement in the tensile strength and cell density of PBAT/PHBV composites made through the injection molding technique. Their results also showed that composite foams made with a combination of N_2_ and CO_2_ physical blowing agents had low quality surface properties compared to those made from expandable thermoplastic microspheres chemical blowing agents. The low solubility of physical blowing agents such as CO_2_ and N_2_ in PHBV has limited the ability to initiate cellular nucleation in the polymer. To this end, researchers have blended it with polymers with low crystallinity in order to foam it. Despite this, immiscibility and phase separation has been reported to occur with an increase in PHBV concentration. This was demonstrated by Richard et al. [35] who made low density PLA/PHBV foams using the batch process with subcritical CO_2_ gas. From their results, they observed that immiscibility in the binary blend occurred at 25 wt.% concentration of PHBV. This was attributed to the high crystallinity of the blend. Additionally, the presence of PLA in the composite was found to be unaffected by the crystallinity of PHBV, which supported the hypothesis of the occurrence of immiscibility of PLA in PHBV. The use of high temperature and pressure conditions have also been explored to achieve cellular nucleation in PHBV. An example of this was the work documented by Xu et al. [38]. They were able to achieve microcellular structures with pore sizes ranging from 6–22 µm when PHBV sheets were foamed with supercritical CO_2_ using foaming temperature and pressure ranges of 145–165 °C and 10–29 MPa respectively. Blending PHBV with less brittle, low melting point thermoplastic polymers like polycaprolactone, a polymer with a high miscibility in CO_2_, might be the solution to developing more miscible binary blends from the polymer [25]. 

Polycaprolactone (PCL), which is also a semi crystalline and hydrophobic polymer, has a high CO_2_ miscibility and has been blended with several polymers. Foams have been produced with filler materials such as calcium phosphate and graphene oxide and foamed into porous structures for use as scaffolds in bone engineering [39,40]. Furthermore, PCL has been used extensively for other pharmaceutical applications especially when blended with biopolymers such as cellulose acetate butyrate and cellulose propionate. These blends have been reportedly foamed and manufactured into large implants and devices that are used for controlling drug release in humans [41]. The extensive biomedical use of PCL for these various applications is due to its superior rheological and viscoelastic properties compared to many of its resorbable-polymer counterparts. This makes it easy to manufacture and deploy as porous structures and degradable implants that can be easily manipulated and tailored to suite specific anatomical sites [42,43,44]. Although synthesized from crude oil, PCL degrades easily along enzymatic and hydrolytic pathways, making it biodegradable and eco-friendly [45,46]. It has been extensively blended with both miscible and immiscible polymers, due to the ease with which its carbonyl groups form intermolecular hydrogen bonds with the hydroxyl groups of many secondary polymers. So far, PCL has been reported to form miscible blends with only a few polymers. These are polyvinyl phenol, poly vinyl chloride, poly vinyl alcohol, poly vinyl methyl ether, bisphenol-A- type epoxy resin, and poly para-chlorostyrene [47,48]. Thermal analysis of solid composites made by blending PCL with each of these polymers has shown miscibility. This is in addition to an absence of melting point peaks and evidence of a constant glass transition temperature, suggesting the occurrence of interdiffusion between the amorphous phases of PCL and the polymers [49,50]. For example, Lezcano et al [47], in their work, reported that PCL formed miscible blends with poly(4-hydroxystyrene) (P4HS) in the molten state due to interactions between the hydroxyl and carbonyl groups at 170 °C [47]. Similarly, Ayse et al. [48] reported miscibility when up to 90 wt. % PCL was melt blended with poly para-chlorostyrene. Investigation of the thermal properties of the blends using the differential scanning calorimeter revealed a single glass transition temperature. Additionally, by studying the viscosity of the individual polymers and the blends and the carbonyl stretching of PCL in poly para-chlorostyrene using Fourier transform infrared spectroscopy (FTIR), miscibility was confirmed. Some researchers have also documented the occurrence of lower critical solution temperature (LCST) behavior leading to phase separation when polymer blends containing PCL were heated above their melting point [50,51]. PCL is ductile and widely compatibility with many polymers, however, it possesses poor mechanical properties. This has limited its use in load bearing applications [52]. Hence, to compensate for this, PCL has been blended with many other polymers and fillers [52,53]. Reports of the mechanical, thermal, and morphological properties of porous composites made by blending PCL with different polymers and fillers have been documented. Evlashin et al. [39] reported an improvement in mechanical properties of PCL/PCL/graphene oxide foams made with CO_2_ as the blowing agent. Results obtained through the digital image correlation system and fatigue tests showed that the inclusion of graphene oxide improved the flexibility of the foams, enabling them to withstand up to about 10^5^ loading cycles. Nano-hydroxyapatite-PCL foam blends made through the microwave processing technique were also reported to show improved tensile strength and hardness values of up to about 145% and 96% for foams with 20 wt.% hydroxyapatite fractions compared to pure PCL foams [50]. Similarly, Huang and Yang [54] achieved stable mechanical properties due to a reduction in the degradation of PLA when PCL/PLA composite blends were made in varying ratios and annealed at low temperatures in sc-CO_2_ to produce biocompatible composites. Results for the thermal properties of PCL were shown to differ depending on the type of polymer or filler it was blended with. Botlhoko et al. [55] melt blended varying concentrations of PCL/PLA and reported a thermogravimetric analysis that showed a two-step degradation process that corresponded to the respective content of each polymer. Maximum thermal stability recorded in the 60% PLA/40% PCL blend confirmed the immiscibility of both polymers as reflected in their morphology. Salerno et al. [56] also reported double melting peaks when PCL foams with 5 wt.% hydroxyapatite inclusions were made through the melt blending technique and foamed with sc-CO_2_ within a temperature range of 37–40 °C and a pressure of 20 MPa for a time range of 1.5–12 h. Results obtained showed a reduction in the melting peaks of the foamed composites compared to the unfoamed ones. This was attributed to the plasticization effect of sc-CO_2_ on the foam blends as reported widely in literature [57,58,59]. Similarly, dual melting peaks were also recorded at 25 wt.% and 33 wt.% mater-bi concentration when PCL/mater-bi foams blends were made at varying concentrations using the sc-CO_2_ batch foaming process. Similarities between the melting enthalpies of both the foamed and unfoamed composites suggested that CO_2_ did not have much effect on the miscibility of the blends [60]. Furthermore, different types of cellular morphologies have been reported for PCL blend of foams. Low density closed cell foams with increased percentage porosity have been made from melt blended talc and PCL using the batch process, while majorly open cell foams were reported for PCL and mater-bi blends [60,61,62]. The development of PCL/PHBV blend of foams is expected to have a wide range of applications because of the special properties of the individual polymers in the blend. It is worthy to note that there is currently no documented information on PCL/PHBV composite foams made through the sub-critical CO_2_ batch foaming process, which typically show higher mechanical performance than CO_2_ foaming of polymer melts. Apart from Qui et al. [63] who studied the miscibility and crystallization behavior of PHBV/PCL blends made through the solvent casting process using chloroform and recorded no miscibility between both polymers, the most significant work done on these binary polymer blends was prepared through the melt mixing and extrusion foaming by Jenkins et al. [25]. In their work, they blended PCL with PHBV, having 8% hydroxy valerate content at a temperature of 125 °C and pressure of 32 MPa for 2 h in the presence of CO_2_. Their results showed that melt blending both polymers led to immiscibility but when blended in the presence of sc-CO_2_, mixed phase spherulites were created. This was attributed to the enhanced interfusion and dissolution of CO_2_ in the polymers and was further supported with the Flory Huggins theory [25,64,65]. 

We have previously utilized PCL with starch copolymers (Mater-bi) for CO_2_ foaming [60]. The foams were aided by close melting temperatures between the two polymers. In this paper, we explore blending PCL with PHBV to foam PHBV. We utilize a two stage depressurization approach to aid nucleation and growth in the foam. We examine the level of dissolution of PCL in PHBV in the unfoamed system and the effect of foaming on the thermal transitions. We then explore the mechanical, thermal, and acoustic performance of the blends.

## 2. Materials and Methods 

### 2.1. Materials

Polycaprolactone (PCL) with a brand name of CAPA 6800 with average molecular weight of 84,000 (g/mol), density of 1.09 (g/cm^3^), melting temperature of 60 (°C), and melt index of (3 g/10 min) was supplied by Perstorp Capa, Malmo Sweden. Polyhydroxy butyrate-co-valerate (PHBV) with brand name ENMAT Y1000P, with approximately 3 mol% of hydroxy valerate was supplied by Ningbo Tainan Biological, China. It had a T_m_ range of 170–176 °C, molecular weight of 240 kDa, and density of 1.19 (g/cm^3^). The solid composites and foams were represented by X_BV and X_BVf, respectively, with “X” representing the percentage weight concentration of PHBV in the blend. X = 0 (0 wt.% of PHBV, 0 vol %), (5 wt.% of PHBV, 4.6 vol%), (15 wt.% of PHBV, 13.9 vol%), (25 wt.% of PHBV = 23.4 vol%), (35 wt.% of PHBV = 33 vol%), (45 wt.% of PHBV = 42.8 vol%), (55 wt.% of PHBV = 52.8 vol%), (65 wt.% of PHBV = 62.9 vol%), (75 wt.% of PHBV = 73.3 vol%), (85 wt.% of PHBV = 83.8 vol%), and (100 wt. % of PHBV = 100 vol%). 

### 2.2. Materials Preparation

**Figure 1:**
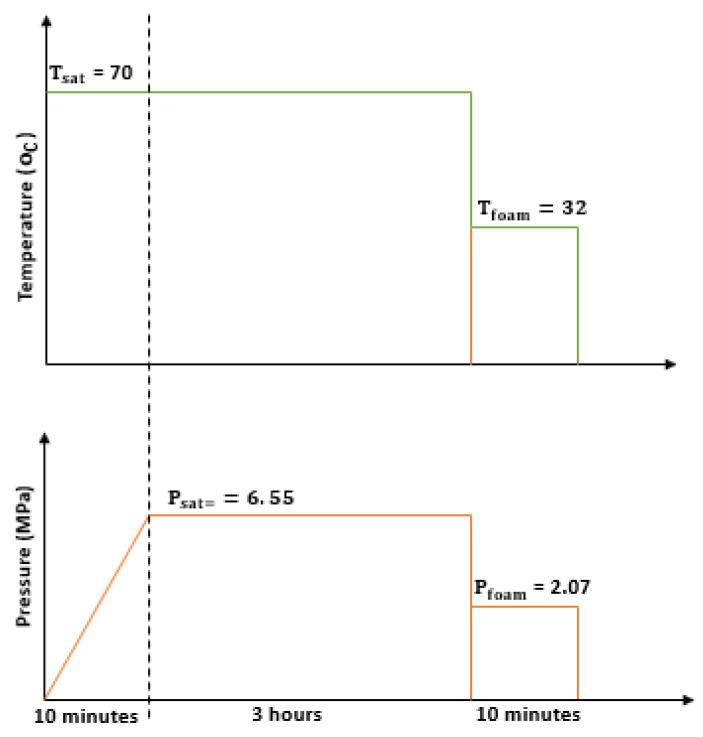
Schematics of the foaming process for PCL/PHBV foams.

The PCL and PHBV pellets were dried in a vacuum oven for over 6 h at 35 °C to reduce moisture. PCL/PHBV blends were made using a Brabender Prep-Center mixer at working temperature and speed of 180 °C and 60 rev/minute respectively for 12 min. This was followed by pelletizing the solidified blend with a Fritsch pelletizer. Compression molded samples were made from the blends using the Carver hot press and subsequently subjected to foaming. Foaming experiments were performed using the two-step decompression technique and the solid-state foaming process. The foaming conditions (Figure 1) used in this work were based on preliminary experiments [61,62]. The pressure vessel containing the samples was pre-heated to a saturation temperature (T_sat_) of 70 °C and allowed to stabilize for 10 min before CO_2_ was released into it. The soaking process lasted for 3 h at a pressure (P_sat_) of 6.55 MPa. After the soaking process, foaming was initiated by cooling and depressurizing the vessel down to 32 °C and a pressure of 2.07 MPa to provide the driving force for cellular nucleation. After these, cell growth was promoted by leaving the samples in the vessel at the foaming temperature for 10 min (t_foam_). This was followed by a second stage depressurization after which the pressure vessel was simultaneously cooled to ambient temperature. The foams were then removed from the vessel and prepared for characterization. As a result of the increasing difficulty experienced in achieving cellular nucleation at higher PHBV concentrations, foaming of the blends was stopped at the 65 wt.% PHBV concentration.

### 2.3. Differential Scanning Calorimetry

A Perkin Elmer DSC 6 differential scanning calorimeter equipped with a Perkin-Elmer Chiller was used for DSC measurements using nitrogen as the purge gas. Heating scans of the samples were performed at 10 °C per minute from 30 °C to 190 °C, held isothermally for 5 min, and then cooled down from 190 °C to 30 °C at the same rate. The weights of the samples were maintained within a range of 5 and 12 mg. Heating and cooling runs were performed twice on each sample. Melting temperatures, crystallization temperatures, melting enthalpy, and heat of fusion were calculated from the heating scans [66]. 

### 2.4. Foam Density and Porosity Analysis

The solid composite and foam densities ρ_p_ and ρ_f_ were determined using ASTM (D1505-98) and (D1622-98) standards [67]. The expansion ratio of the foams was calculated as the ratio of densities of unfoamed polymer to that of the foams using Equation (1) below [68].

(1) $$\mathrm{Expansion}\;\mathrm{ratio}=\frac{\rho_{p}}{\rho_{f}}$$

Percentage open porosity and the cell type of each of the foams was obtained using the Ultrapyc 1200e model pycnometer made by Quantachrome Instruments Inc. Its working principle was based on Boyle’s law and the Archimedes principle of displacement of fluids in a porous media. Helium gas was the operating fluid used with the pycnometer [69,70].

### 2.5. Scanning Electron Microscopy (SEM)

The morphology of the cross-section of prepared samples was examined using an environmental scanning electron microscope (FEI Quanta 200 ESEM). The foam samples were initially freeze-fractured in nitrogen after which they were coated with a thin film of Gold/Palladium to make them conductive to obtain a clear image of the cross section of the PCL/PHBV foams. An accelerating voltage of 5 kV and working distance of 10 mm were used for the analysis [71].

### 2.6. Foam Morphological Analysis

The number of cells (bubbles) in each of the micrographs obtained from the SEM was counted using the Image J Pro software. Between 100–200 cells were present in each micrograph. For greater accuracy, cells (*n*) were counted in four different sections of each micrograph and the area (A) was calculated in centimeter square, where M is the magnification. From these, the cell density (N_f_) for each of the foams was calculated using Equation (2) below [70].

(2) $$N_{f}={\left(\frac{{\mathrm{nM}}^{2}}{A}\right)}^{3/2}\times \mathrm{Er}$$

The cell size for the foams was calculated using Equation (3) below [72].

(3) $$N_{s}={\left(\frac{\mathrm{Er}-1}{N_{f}}\right)}^{1/3}$$

Void fraction (V_f_) for the foams was calculated using Equation (4) below [73].

(4) $$V_{f}=\left(1-\frac{1}{\mathrm{Er}}\right)\times 100$$

Cell wall thickness of the foams was calculated using Equation (5) below. Where C_wt_ is the cell wall thickness, C_s_ = cell size, ρ_f_ = density of foam, and ρ_p_ = density of unexpanded polymer [74].

(5) $$C_{\mathrm{wt}}=N_{s}\times \left(\frac{1}{\sqrt{1-\frac{\rho_{f}}{\rho_{p}}}}-1\right)\;$$

### 2.7. Acoustic Properties Measurement

The acoustic performance of the composites in terms of sound absorption and acoustic impedance were obtained by using a two-microphone impedance tube. All measurements were done in accordance to ASTM E1050-12 standard [75]. The acoustic properties were measured for both solid and foam composites using cylindrical samples with diameter of 38 mm and thickness of about 15 mm over a frequency range of 80–5000 Hz. The incident sound wave was normal to the surface of the foam rise direction. Each of the tests was repeated three times to obtain consistent and representative results. Average and standard deviation were then computed from the readings [76]. While microstructural and non-acoustic properties of polymer composites foams do not depend on the size of the sample, sound absorption coefficient and surface impedance mainly depend on the sample thickness [77]. To minimize variation in the results obtained, the thickness of the samples was kept constant throughout the experiment.

### 2.8. Thermal Conductivity Measurement

To determine the thermal insulation performance of the composites, thermal conductivity property was measured at room temperature using a Hot-Disk thermal constants analyzer (TPS 1500) made by ThermTest Inc. Dimensions of 50 mm diameter by 20 mm thickness were used for both the unfoamed and foam samples. The Kapton sensor (with Ni spiral for heating), which was used as both the heat source (at 0.012 W power rating in the measurements) and temperature sensor, was sandwiched between two identical samples. The transient heat conduction test was carried out in an isotropic dual mode for 160 s. Thermal conductivity was calculated automatically by the instrument using the transient heat diffusion equation. To account for possible variation in heat flow rates across the foams based on the difference in morphology, tests were carried out from the foam’s exterior to its core. Three replicates of each foam sample type were tested. To minimize variation and ensure a high degree of accuracy in the results obtained, each experiment was repeated six times at 15 min interval for each sample, making a total of 18 tests per sample. The mean effective thermal conductivity (${ƛ}_{T}$) value and standard deviation of each sample set were subsequently determined. The mean effective thermal conductivity (${ƛ}_{T}$) of the foams could be represented by Equation (6) below [78,79].

(6) $${ƛ}_{T}={ƛ}_{\mathrm{sol}}+{ƛ}_{g}+{ƛ}_{r}+{ƛ}_{\mathrm{cv}}$$

where ${ƛ}_{sol}$ = conduction through the solid phase, ${ƛ}_{g}$ = conduction through the gas phase, ${ƛ}_{r}$ = thermal radiation, and ${ƛ}_{\mathrm{cv}}$ = convection in the gas phase = 0 (since the cell size of the foams are <4 mm).

### 2.9. Mechanical Testing (Compression)

Measurement of compressive mechanical properties of the microcellular foams was performed on a Shimadzu AG-X plus series machine in compression mode at room temperature. Compression tests on the unfoamed samples were conducted on a 30 mm diameter disc having a 25 mm height, while foams were tested using a sample that had a diameter of 50 mm and thickness of 30 mm. The testing tool and machine was configured using guidelines in ASTM D695 for the unfoamed plastic composites and ASTM D3574/D3575 for the foamed composites respectively [80]. A crosshead speed of 1.3 mm/min was used to perform the compression tests, and loads of 10 KN were applied to both the unfoamed and foamed composites. From the stress and strain curves, values of compression modulus and compression strength were determined. 

## 3. Results 

### 3.1. Foam Morphology

The morphological properties, which include expansion ratio, percentage porosity, void fraction, cell density, size, and wall thickness of the foams are shown in Table 1. The expansion ratio, which is calculated as the ratio of densities of unfoamed polymer to that of the foams, shows that as the PHBV concentration increases, the expansion ration decreases [79]. The decreasing expansion ratio with increasing PHBV presence impacts the porosity, and three regimes are evident. First the 5% PHBV showed an increase in porosity and expansion ratio over the pure PCL. This reflected that PHBV acted as an effective nucleating agent within the PCL matrix, leading to a 7 wt.% increase in foam expansion ratio when compared to pure PCL foam. This could be attributed to the low melt strength and viscosity of the composites with low PHBV weight concentrations. The rupturing of their delicate cell walls during the foaming process produced open cell foams with high void fraction due to the low melt strength of the composites with high PCL concentration [81]. However, as the concentration of PHBV in the blend increased, the expansion ratio decreased, reflecting the impact of low CO_2_ solubility in PHBV. Between 15 to 35%, a porosity greater than 50% was observed through the use of a pycnometer [82]. As PHBV increased, between 45 to 65 we found that the expansion ratio reflected minimal expansion in the foam and porosity fell below 50%. 

**Figure 2:**
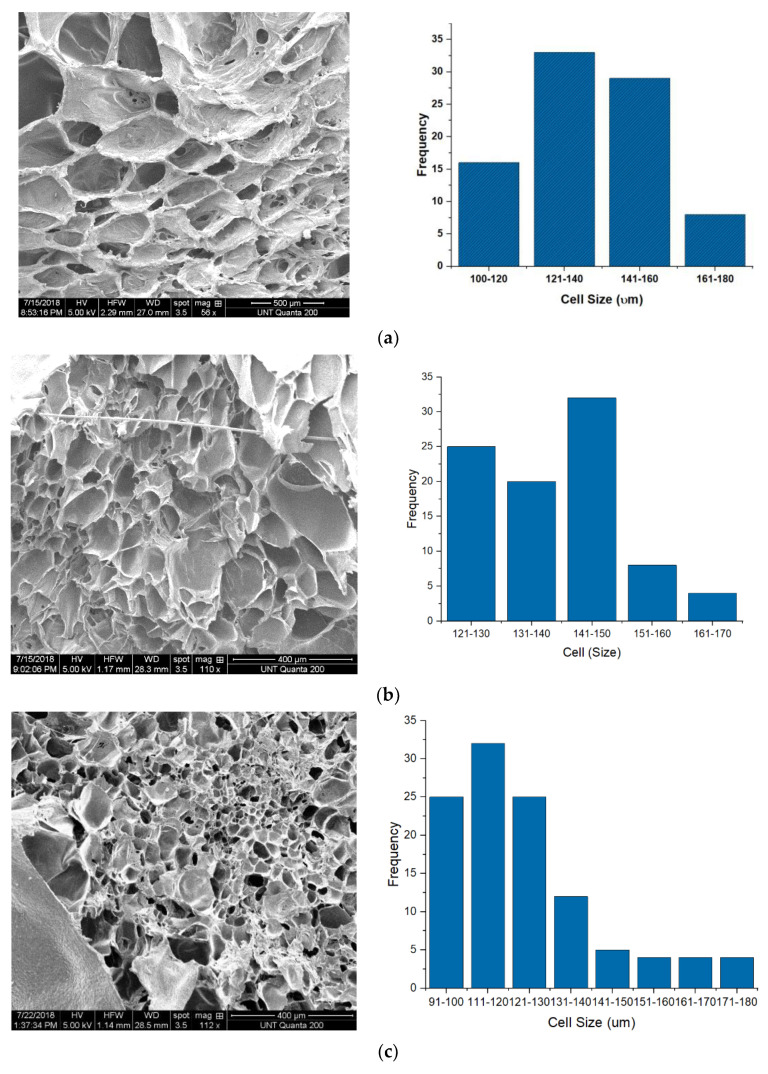

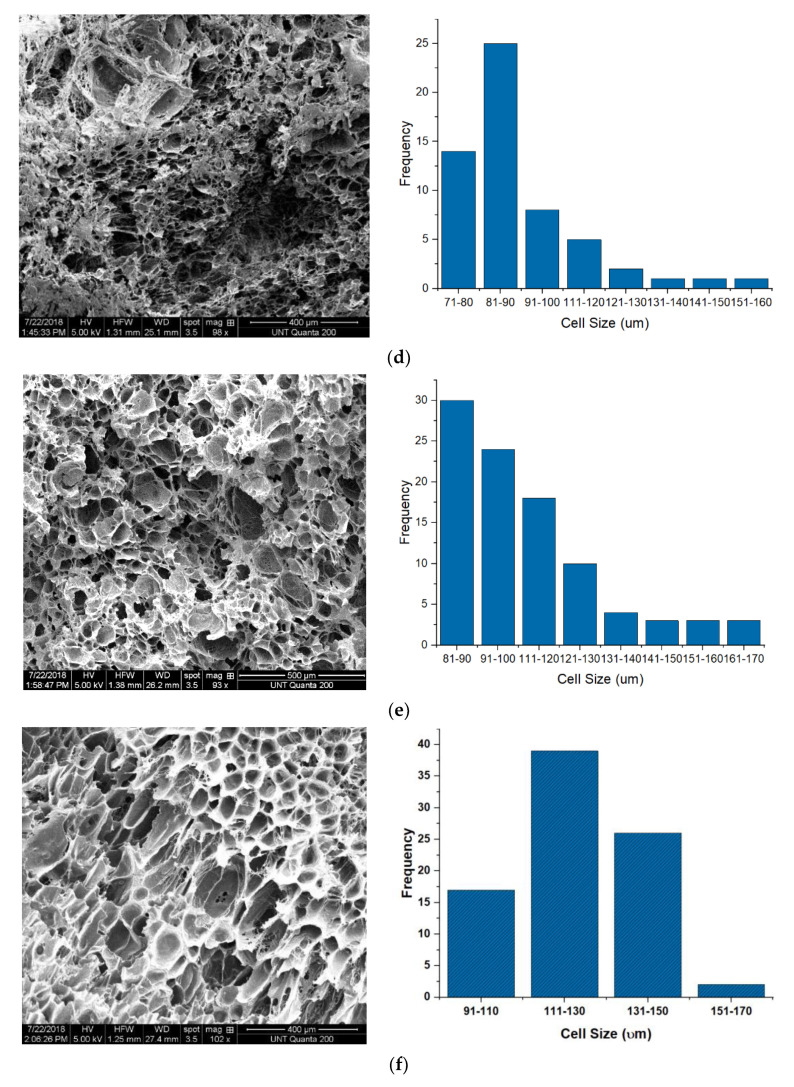

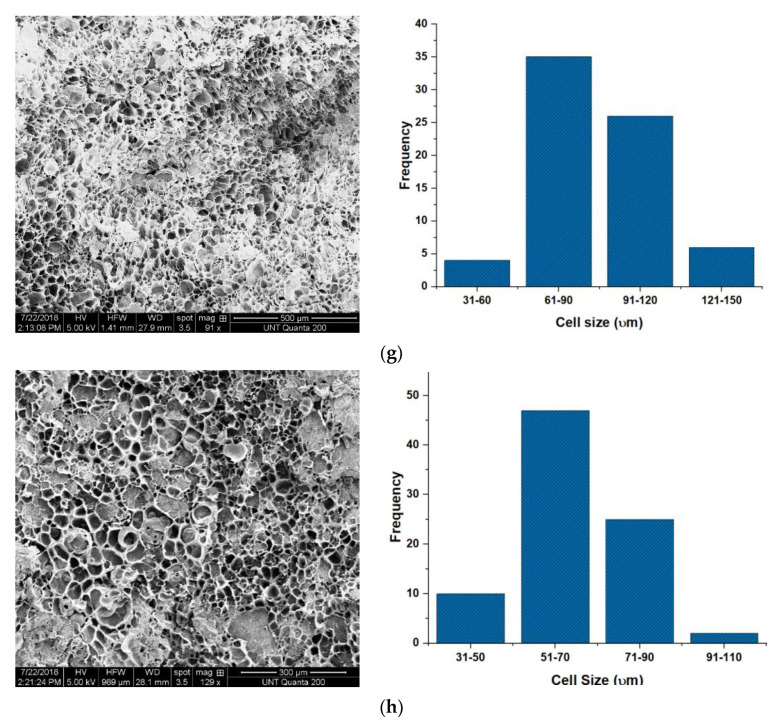
SEM micrograph and cell size vs. frequency distribution: (**a**) 0_BVf, (**b**) 5_BVf, (**c**) 15_BVf, (**d**) 25_BVf, (**e**) 35_BVf, (**f**) 45_BVf, (**g**) 55_BVf, (**h**) 65_BVf.

Visual observation of the foams showed that that the coloration of the foams transitioned from white to cream then from cream to tan as the concentration of PHBV increased within the blend. SEM imaging was used to observe the cellular microstructure for each of the foam blends (Figure 2a–h). ImageJ analysis of cell sizes was carried out on the SEM micrographs and used to quantify the cell size and cell densities of the foams. The SEM imaging showed that at very low PHBV concentrations, the foam structure initially possessed large uniform polygonal honeycomb cells, which became a mixed cellular structure of small and large dual open/closed cells at increased concentrations, which then later transitioned into small spherical closed cells at PHBV concentrations beyond 45 wt.% (Figure 2a–h). Similar results have been reported for PCL/talc foams [62].

**Figure 3:**
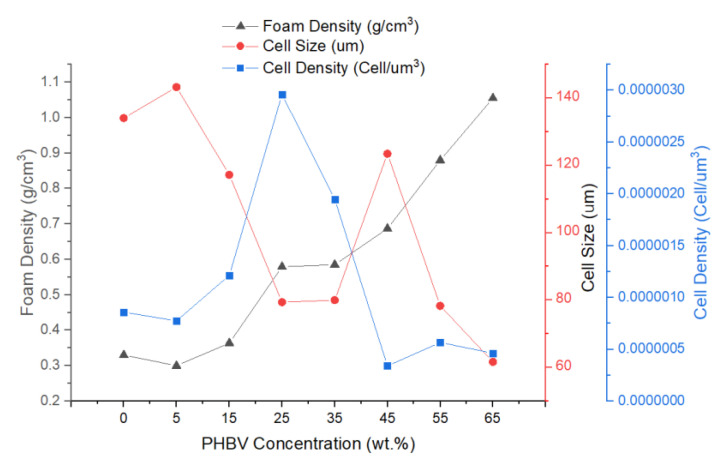
Effect of PHBV concentration of the cellular morphology of the PCL/PHBV foam blends.

From the micrographs, in the foam blends with (15–35) wt.% PHBV, a mixed cellular morphology, a narrow range of cell densities with cell sizes that reduced as PHBV concentration increased was observed (Table 1). Furthermore, the dual open/closed cell morphology present in the 35 wt.% PHBV foam possessed a 50.25% open porosity and 54% void fraction, which suggests that maximum stretching of the melt occurred in this foam fraction and indicates that this foam fraction is the terminal point of open porosity. Figure 3 below shows the effect of PHBV concentration of the cellular morphology of the foam blends.

### 3.2. Differential Scanning Calorimetry (DSC)

**Figure 4:**
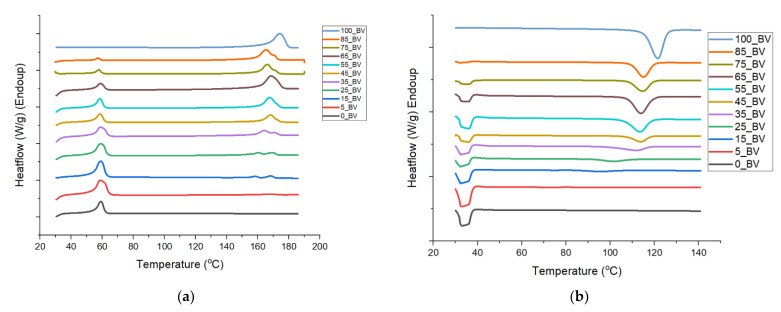

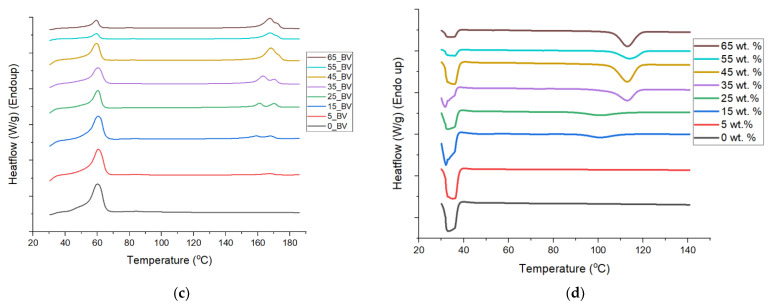
DSC plots of unfoamed and foam PCL/PHBV composites: (**a**) Second heating cycle (unfoamed); (**b**) First crystallization cycle (unfoamed); (**c**) First heating cycle (Foams); (**d**) First crystallization cycle (Foams).

The DSC of the melting and recrystallization of the pure polymers and blends (unfoamed and foams) are shown in Figure 4 and tabulated in Table 2 and Table 3. A glass transition of −60 °C and −1 °C, which was determined for PCL and PHBV, respectively, was not distinguished for blends with a breadth ranging from −60 to ~90 °C, and thus we focus on the melting DSC analysis. Examining the second heating scan to erase the thermal history in the unfoamed systems, revealed that the peak melting points of pure polymers PCL and PHBV were 58.35 °C and 174.41 °C respectively (Table 2). In the unfoamed composites, it was observed that as the concentration of PHBV increased in PCL, the melting point for the PCL rich phase did not change significantly but showed only a narrow range of T_m1_ values (57.1–59.47) °C. However, as PHBV was introduced into PCL, variances with respect to the pure PCL peak enthalpy were observed, reflecting disruption of the PCL crystallite by the PHBV presence. 

A significant increase (53%) in the enthalpy of melting corresponding to the PCL phase when PHBV was first introduced into PCL at 5 wt.% indicates a high PHBV integration into the PCL crystallite. As PHBV weight concentration increased from (5–35) wt.%, a decrease in H_m1_ values was observed relative to the 5_BV, while higher than pure PCL, which indicates continued dissolution of PHBV in PCL. In this range, a splitting of peaks for the PHBV phase was observed (Figure 4a). The split indicates a peak closer to the pure PHBV melting peak at 174.1 °C denoted as T_m3_ and one closer to the PCL phase was denoted as T_m2_. T_m2_ represents the region of interaction between the PCL and PHBV phases. Initially the peaks for 15_BV are 158.71 °C and 168.2 °C, while for 35_BV the values are 164 °C and 171 °C, which indicated that as more PHBV was added, the values moved higher towards the pure PHBV melting point of 174.1 °C. For the PHBV range between 45 and 65 wt.% concentration, the unfoamed blends indicated no change in the PCL peak, but the PHBV peak was singular and trended towards the pure PHBV phase. For 75_BV and 85_BV with the majority phase being PHBV, it can be noted that peak splitting reoccurred. 

The recrystallization cooling thermograms (Figure 4b) in the unfoamed blends reflect two recrystallization temperatures, T_c1_ for PCL and T_c2_ for PHBV. At 5 wt.% PHBV concentration, an increase in enthalpy of crystallization for peak T_c1_ was recorded compared to that of pure PCL, which paired to a large drop in the PHBV recrystallization peak from 121 °C in the pure polymer to 73.2 °C. This supports a high dissolution of PHBV into the PCL crystallite at 5% concentration PHBV. The results at other concentrations between 15 and 85% PHBV show that the PCL peak was largely unaffected from the pure polymer, but the PHBV peak increased towards the pure PHBV peak. However, with all blend recrystallization temperatures of the PHBV phase being lower than the pure PHBV, one can infer that PCL had disrupted PHBV crystallites and reduced the crystallite density reflected by both the decrease in temperatures and enthalpy of recrystallization.

**Figure 5:**
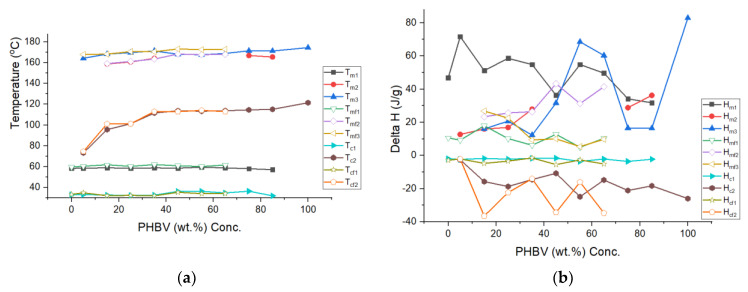
Plot of temperature-enthalpy vs. PHBV concentration: (**a**) All temperatures and (**b**) All enthalpies.

The corresponding foam blends show some distinctions from the pure systems. First the PCL melting temperatures show that the narrow range of (59.6–61.94) °C for melting peaks T_m1_ suggests that a similar crystalline structure existed between pure PCL and the foamed blends (Table 2). The PCL melting curves show similarities between the unfoamed and foamed blends for all compositions in temperature with a substantial drop in melting enthalpies indicating the CO_2_ reduced the crystallite density in the PCL both in the pure and blended systems. The PHBV phase in all foams showed peak splitting in all compositions that were foamed. All temperatures were slightly higher in the foam blends than in the unfoamed blends and all enthalpies were much lower in the foams over the unfoamed polymers. This is shown in Figure 5a for the temperature overlay and in Figure 5b for the enthalpy overlay.

### 3.3. Acoustic Properties 

**Figure 6:**
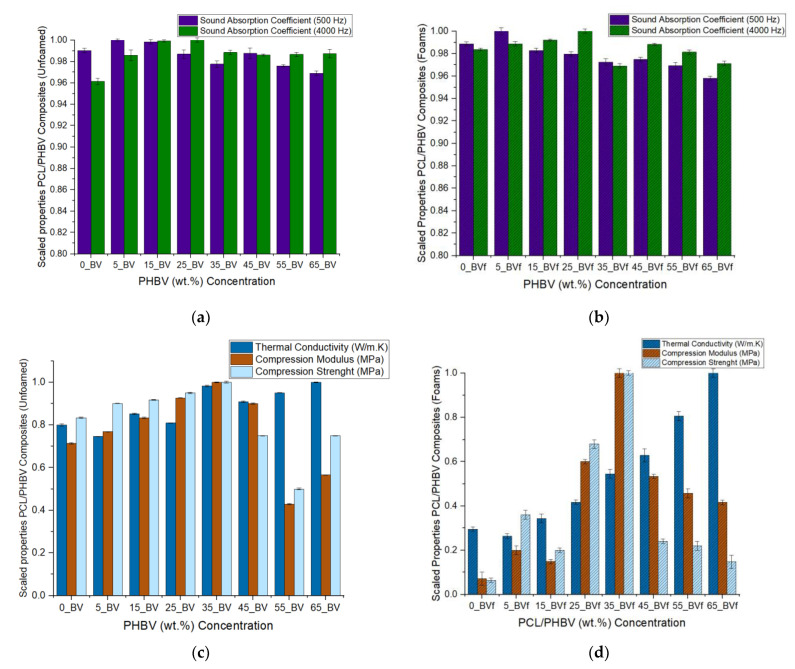
Scaled values for performance properties PCL/PHBV blend composites: (**a**) Acoustic properties for unfoamed blends; (**b**) Acoustic properties for foams; (**c**) Thermal and mechanical properties for unfoamed blends; (**d**) Thermal and mechanical properties for foams.

**Figure 7:**
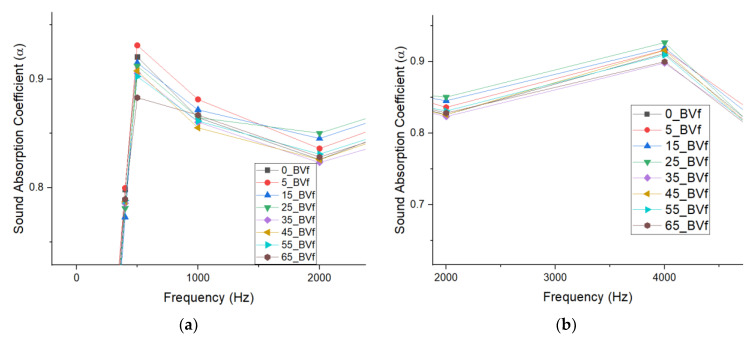
Plot of Sound Absorption coefficient vs. frequency for foams (Core): (**a**) Low Frequency (500 Hz); (**b**) High Frequency (4000 Hz).

The sound absorption results recorded for the binary blends show that improvements in acoustic properties occurred in the unfoamed composites compared to pure PCL (Table 1). For instance, about 1% and 2.5% improvement in the sound absorption coefficient was recorded for the 5 wt.% PHBV unfoamed blend at both low and high frequencies respectively compared to pure PCL. For the foams, cellular morphology, specifically cell size, cell density, porosity, and interconnectivity of cells was observed to have significantly impacted their acoustic properties at different frequencies [83]. This can also be seen in the scaled plot, which shows minor improvements in sound absorption properties of the foam blends when compared with the unfoamed blends (Figure 6a,b). To keep all parameters constant and eliminate variation, acoustic testing was carried out only in the core of the foams for both the open cell and closed cell foams. Acoustic impedance, which is a foam internal structural factor, was suspected to be largely controlled by the cell size and density of the PHBV foams. Two of the open cell foams showed maximum sound absorption properties at two different frequency levels. The 5 wt.% PHBV foam was best at low frequency of 500 Hz, while the 25 wt.% PHBV foam was best at a high frequency of 4000 Hz (Figure 7a,b). This could be attributed to the large cell size and low foam density displayed by the 5 wt.% PHBV foam and the large air volume contained in the 25 wt.% PHBV foam as shown by its large cell density in Table 3 [83,84,85].

Similarly, for the closed cell foams, about 0.5% improvement in sound absorption coefficient was recorded for the 45 wt.% PHBV foam at high frequency compared to the 0% PHBV foam. For both the open and closed cell foams, it was suspected that the arrangement of the crystallites at these PHBV concentrations and the presence of the mixed phase (T_m2_), which was earlier stated to have influenced the cellular morphology of the foams, were responsible for the improvement in their sound absorption properties. For the 25 wt.% PHBV foam, bulk absorption occurred in it due to a higher CO_2_ miscibility as suggested by its open cell structure, while only surface adsorption of CO_2_ occurred at 45 wt.% PHBV due to immiscibility and closed cell nature. Both phenomena resulted in the highest cell density and maximum sound absorption properties at high frequency recorded by both foams in the open cell and closed cell foam categories, respectively (Table 1).

From the enlargement of the sound absorption plot in Figure (Figure 7a) above, it was observed that all the foams showed a lower sound absorption coefficient below 500 Hz. This was suspected to be due to the smaller magnitude of the foam sample thickness compared to their individual acoustic wavelengths (λ) as shown in Equation (7) below.

(7) $$\lambda =\frac{c}{f}$$

where *c* = particle velocity (m/S), and *f* = frequency (Hz). 

**Figure 8:**
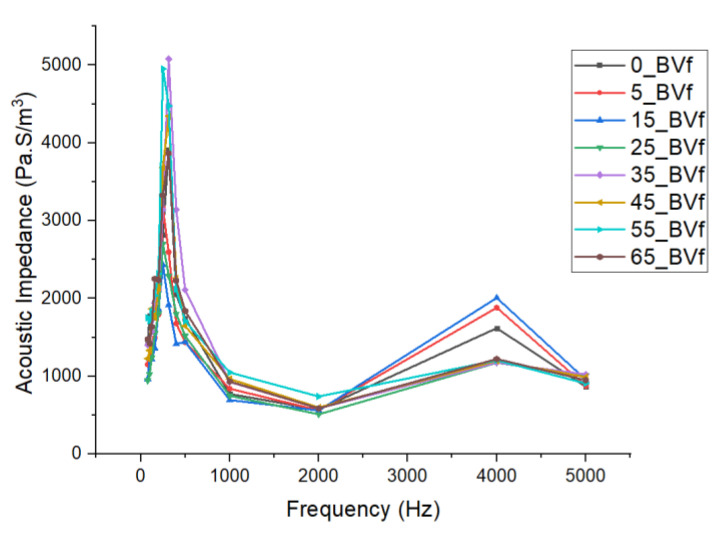
Plot of acoustic impedance vs. frequency for the composite foams.

This phenomenon described above suggested that below 500 Hz, acoustic energy in the foam pores was not sufficient to lead to rapid energy dissipation. However, at 500 Hz, an increase in dissipation occurred, leading to higher sound absorption. Similarly, the dip in absorption coefficient recorded between 1000 Hz and 2000 Hz was suspected to be due to an increase in the imaginary part of the impedance. However, at frequencies beyond 3000 Hz, the wavelength became close in magnitude to the foam thickness, leading to higher energy losses and increased absorption as a result of greater interaction between the incident sound waves and the internal structure of the foams [76]. At about 4000 Hz, it was observed that the sound absorption coefficients recorded for the foams were directly related to their characteristic acoustic impedance (Figure 7b). In addition to the influence of its large cell size, about 1.2% improvement in sound absorption coefficient recorded for the 5 wt.% PHBV foam compared to the 0 wt.% PHBV foam at low frequency could be attributed to its high void fraction and percentage porosity (Table 1). With a cell diameter that is about 7% larger than that of 0 wt.% foam, the higher volume of air in the pores improved absorption at low frequency by rapidly dampening incoming sound waves due to the Helmholtz resonance effect. This facilitated the rapid absorption of the sound pressure exerted on the foam surface at low frequency. Additionally, superior sound absorption properties showed by the 25 wt.% PHBV foam at high frequency could also be attributed to the presence of interconnected architecture shown in their mixed cellular morphology [21]. At this concentration, the partial miscibility of PHBV in the blend produced a cellular morphology with a symmetrical distribution of the pores within the foam structure, as suggested by the bell curve shown in the SEM image for the foam in Figure 2d above. This symmetry in cell size distribution in addition to its majorly open cell properties shown by the void fraction and percentage porosity of the foam promoted the conversion of the incident sound waves to heat due to friction with the cell wall at high frequency. Furthermore, the large pore size (Ø = 123.48 µm) recorded for the 45 wt.% PHBV foam made it the most effective closed cell foam at low frequency as explained earlier. Conclusively, these results have shown that the acoustic behavior of the porous PCL/PHBV blends especially at low frequencies, which may not totally be a function of their bulk densities [84]. Apart from the influence of cell density on the absorption properties of the open cell foams, their internal discontinuities and extensive mixed cellular morphology impacted their acoustic impedance property. This occurred because of an increase in the tortuosity of the incident sound waves propagated through the foams and rapidly converted them to heat and kinetic energy. The lowest impedance value recorded for the 25 wt.% PHBV foam further suggests that the reduction in particle velocity experienced by the sound wave was due to the complexity of the sound transmission path within the mixed cellular foam structure (Figure 8). The large fluid volume in its cell density further increased this tortuosity and was responsible for the peak sound absorption coefficient recorded for the foam at 4000 Hz.

Additionally, it was observed that as cell density and porosity decreased steadily at increased PHBV concentrations, a resultant increase in acoustic impedance of the foams led to reduced particle velocity. However, the absence of a complex internal structure in these foams with higher percentage PHBV concentrations resulted in lower sound absorption coefficient values recorded for them [84].

### 3.4. Thermal Conductivity 

The thermal conductivities of unfoamed and foam composites are also displayed in Table 1. The thermal conductivity of unfoamed PCL with 5 wt.% PHBV dropped by 6.8% compared to pure PCL, suggesting that PHBV was highly miscible with PCL at low concentrations. The reduction in thermal conductivity values was sustained at low values up to 25 wt.% PHBV concentration and increased at PHBV concentration above 25 wt.%. This trend could be attributed to the agglomeration of spherulites, which occurred at PHBV concentrations beyond 25 wt.% [85,86]. For foams, the lowest thermal conductivity value of 0.076 W/m·K was recorded in the foam blend with 5 wt.% PHBV concentration. This could be attributed to a reduction in interfacial adhesion between the phases comprising the porous blends. Besides the effect of PHBV concentration, the thermal conductivities of the foams was also influenced by porosity and cell wall thickness. The large volume of air in the pores of the 5 wt.% PHBV foam, as shown by its high cell size and void fraction, as well as its thinner cell wall, further reduced the thermal conductivity of the foam matrix, leading to a high thermal insulation performance. However, as PHBV concentration increased, 3D networks were formed, leading to an increase in conduction in the solid polymer phase, which reduced the thermal insulation performance of the foams beyond 25 wt.% PHBV concentration as the mean free path of the phonons increased. 

Simulations were carried out to compare with experimental results for foams. Predefined finite element functions for heat transfer analysis in porous media were applied in COMSOL Multiphysics software version 5.3a. The effective thermal conductivity of the foams was determined using the unfoamed polymer and void fractions as contributors. The basic idea of the model was that each foam system was an isotropic continuous phase system, which was made up of a porous blend of PCL/PHBV filled with random pockets of air. The percentage of void fraction obtained from Table 1 above gave an idea of the total amount of air in each composite. Cylindrical geometry was used for each model, which matched the shape of the foams used in the measurements. The free tetrahedral mesh function in the software was applied to the geometry [87]. The density, thermal conductivity, and specific heat values from Table 1 above were used in the simulation. The specific heat of the solid (unfoamed) composites was measured by the DSC. The specific heat capacity values of the corresponding foams used for modeling were calculated by using the rule of mixtures shown in Equation (8) below [88].

(8) $$C_{p\;\mathrm{mixture}}=\left(mass\;fraction\;of\;polymer\times C_{p\_\mathrm{polymer}}\right)+(mass\;fraction\;of\;air\;in\;void\times C_{p\_\mathrm{air}})$$

where $C_{p\_\mathrm{polymer}}$ = specific heat capacity of the unfoamed polymer (J/Kg·K); $C_{p\_\mathrm{air}}$ = specific heat capacity of air (J/Kg·K). The density and specific heat capacity values of air were 1.23 kg/m^3^ and 1000 J/Kg·K, respectively. 

The theoretical thermal conductivity (${ƛ}_{T}$) of the foams was computed using the correlation in Equation (9) below [89,90]:

(9) $${ƛ}_{T}={Ɵ}_{p}\;{ƛ}_{p}+\left(1-{Ɵ}_{p}\right)\times {ƛ}_{\mathrm{air}}$$

where ${Ɵ}_{p}$ = Volume fraction of the polymer matrix; ${ƛ}_{p}$ = thermal conductivity of the polymer matrix (W/m∙K); and ${ƛ}_{\mathrm{air}}$ = thermal conductivity of air (W/m∙K) = 0.0267. In the simulation, the thickness and radius of the foams were taken as 20 mm and 25 mm respectively, which were like the sample dimensions used for the measurements. For the boundary conditions, heating power of 0.012 W was applied at the top surface of the specimen and the other boundaries of the sample were held in natural convection mode to account for heat losses to the environment. The results in Table 4 below show the mean effective thermal conductivity values across each foamed sample. It shows good agreement between the theoretical predictions Equation (9) and experimental measurements, with the difference ≤ 10%. The foam cell wall thickness and bulk density play the major roles in affecting the thermal conductivity values of the foams.

**Figure 9:**
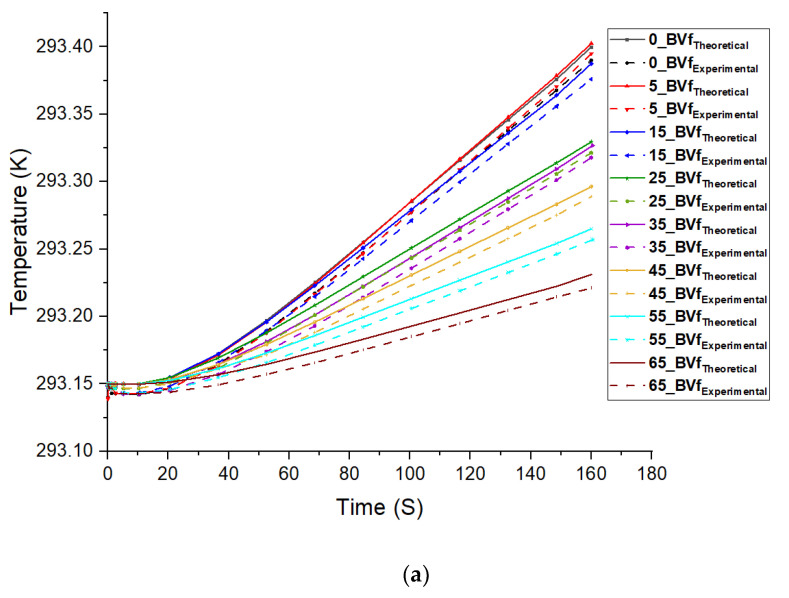

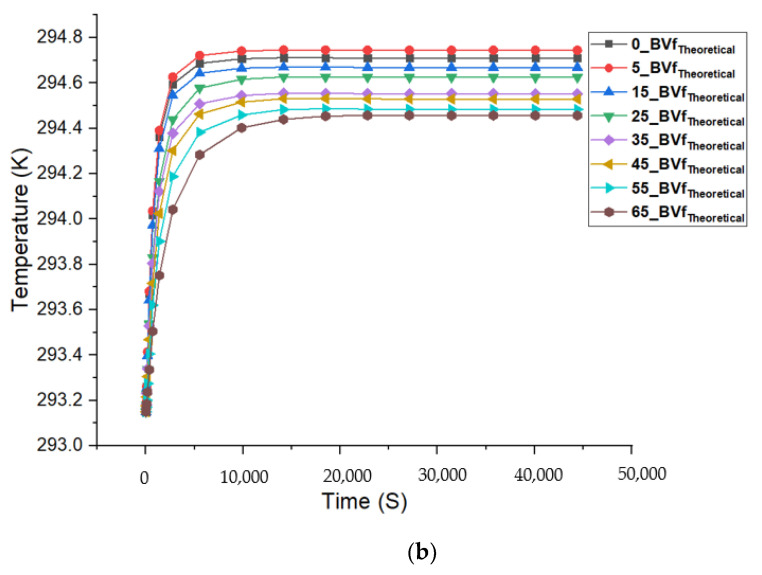
Plot of temperature vs. time for both theoretical and experimental data input for the composite foams (0–65) BVf; (showing temperature profiles at the mid-point of foams) (**a**) for Transient state and (**b**) Steady state.

Figure 9a below displayed the temperature variations in the middle of the foams. The sample temperatures that were modeled based on the theoretical thermal conductivity values from Equation (9) showed good agreements with those based on the measured thermal conductivity value inputs. As seen from the slope of the samples, the temperature of the 5 wt.% PHBV foam with the lowest thermal conductivity value rose faster than that of the other foam samples during the heating process (0.012-W heating power input) because of its good insulation performance, which reduced the heat loss and held the heat inside the foam. This can be attributed to the large volume of air in its pores as seen from its large cell size. Additionally, its large void fraction and porosity compared to the other foams minimized the movement of phonons. This further lowered the heat transfer rate and dissipation within the foam matrix due to its high resistance, which promoted its insulation property. The results of the simulated temperature profile of the foams verified the thermal conductivity mechanism in the foams. Simulations were run for longer periods using the same parameters. This was done to examine the thermal performances of the foams at steady state conditions. As observed in Figure 9b below, the results obtained at steady state also show that the 5 wt.% PHBV foam had the highest internal temperature. Its low foam density and large cell size greatly reduced thermal conductivity, resulting in its higher thermal insulation performance compared to its contemporaries. Furthermore, the open cell and closed cell foams were observed to be clustered together in their individual categories with the 25 wt.% PHBV acting as a boundary line between both foam types. This suggested that the thermal insulation performances of the foams were largely influenced by their cellular morphology.

### 3.5. Mechanical Property

**Figure 10:**
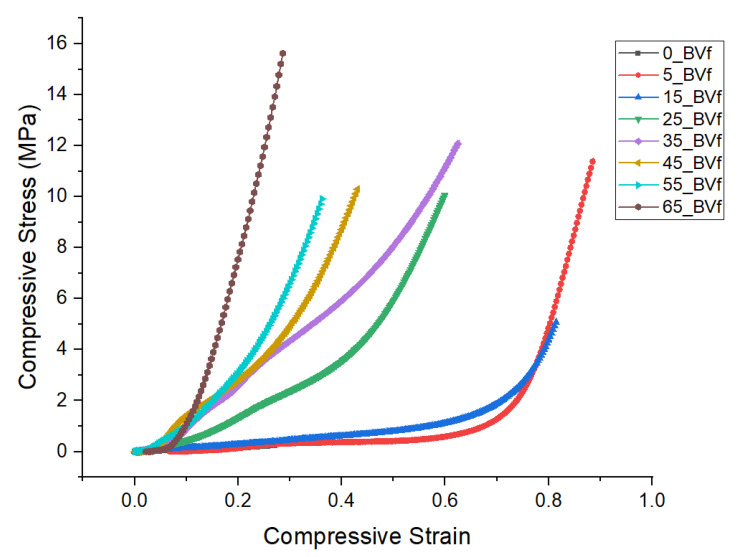
Stress vs. strain curve for the composite foams.

The results shown for compression modulus and strength for the unfoamed composites suggest that the inclusion of PHBV in PCL matrix improved the mechanical properties of the composites. Steady improvement was recorded with increase PHBV concentration, with peak values occurring at 35 wt.% concentration. This signified 40% and 20% improvement in compressive modulus and strength, respectively, compared to the pure PCL sample before declining (Table 1). Similarly, for the foams, maximum compression strength of 2.5 MPa, which was recorded in the 35 wt.% foam blend, suggests that the crystallites were able to provide maximum reinforcement to the cell wall at this concentration. The dual open cell and closed cell morphology exhibited by its 50% percentage porosity property and its mixed cell sizes, were largely responsible for the peak mechanical performance exhibited by the 35 wt.% PHBV foam blend (Table 1). Furthermore, a steady reduction in the mechanical properties shown by the foam blends beyond 35 wt.% PHBV concentration suggested that phase separation due to immiscibility as the crystalline regions fused, led to a decline in the reinforcing abilities of the spherulites in the blend. This implies that mechanical properties of the blends may not have followed a linear function as the PHBV concentrations increased (Figure 10). Additionally, phase inversion and chain mobility, which led to the shearing of atoms during mechanical loading, contributed to the decline in compression strength recorded in the foams with higher PHBV concentrations [60]. Peak mechanical performance of the 35 wt.% PHBV blend is shown in the scaled plot in Figure 6c,d. As shown in the DSC results of the foams, maximum T_mf1_ value of 61.94 °C and lowest enthalpy value of 9.49 J/g recorded for the 35 wt.% PHBV concentration were an indication of peak interaction between PCL phase and PHBV phase. At this point, maximum dissolution of PCL in the PHBV phase, which occurred and was facilitated by the lubricating effect of CO_2_, further enhanced the interlocking of the phases. As a result of this, the simultaneous sequence of nucleation and melt recrystallization led to a rearrangement of the lamellae chains as indicated by the presence of the mixed phase. This was responsible for about 25% and 300% improvement in modulus and strength recorded for the 35 wt.% PHBV foams. Other factors that were responsible for the higher mechanical properties observed in the foams could be attributed to the improved bonding that occurred in the binary blend due to the attraction between the non-polar sc-CO_2_ molecules, the polar heavy ethyl side chains of the valerate (HV) unit, and the side groups in the micelle core of PCL [91,92]. This toughened the crystalline regions in the foams and reinforced the cell walls with a corresponding increase in bulk density [93,94]. 

## 4. Conclusions

**Figure 11:**
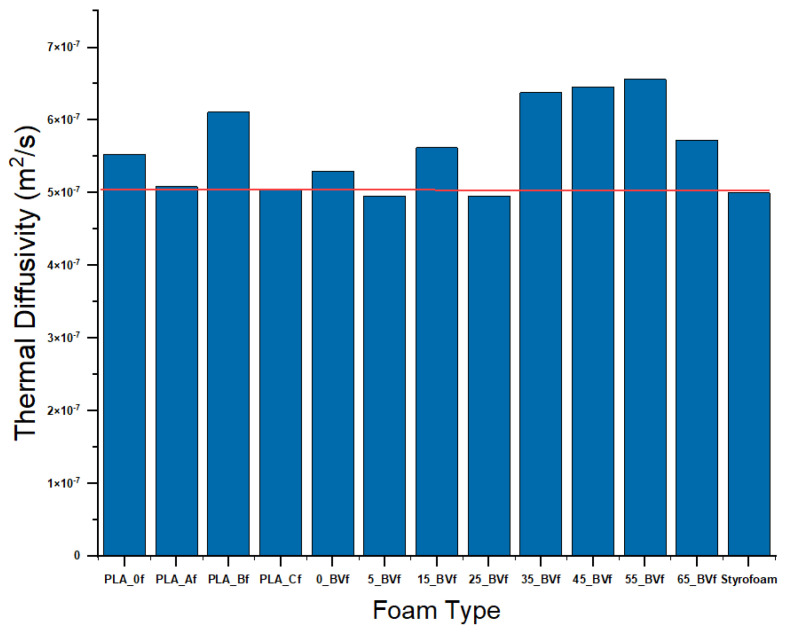
Thermal diffusivity for the foams compared to Styrofoam.

Using CO_2_ to foam a low CO_2_ solid state soluble polymer like PHBV using a highly CO_2_ soluble polymer like PCL was shown to be feasible. The results indicate that the polymer–polymer interactions impact the net propensity of the blend to foam. The ability for PHBV to disrupt the PCL crystallites and for PCL to disrupt the PHBV crystallites resulted in PCL presence, but the magnitude of its impact in the PHBV crystallite affected the ability for the blend to foam. A two-step approach to increase the time for growth of the foam bubble was applied using the two-step decompression technique of the solid-state batch foaming process with sub-critical CO_2_. Cellular nucleation that occurred in the blend was attributed to the ability to achieve good dispersion of the phases through intense Brabender mixing of the blends. The blends reflected three regimes. First, when PHBV was just introduced into PHBV, a disruption of the PCL crystallite led to larger cell sizes than in pure PCL in the resultant foam. For 15–35%, a new mixed crystallite was observed closer to the PHBV pure melt peak. For 45 and above, the system moved closer to pure PHBV crystallite. The extent of PCL disruption of PHBV crystallites led to a gradually decreasing expansion ratio and porosity in the foam with increasing PHBV presence. Pure PCL showed the highest porosity and expansion ratio, reflecting its high CO_2_ miscibility. As PHBV was introduced, porosity gradually decreased with a porosity greater than 50% (indicating open cell architecture presence) recorded in the blends up until 35% PHBV concentration. The expansion ratio correspondingly showed the highest magnitude for pure PCL and gradually decreased. Little to no expansion was obtained above 55% PHBV, reflecting a limited ability for the PCL to be incorporated into PHBV. Performance in the foam was found to be a compounded effect of both decreased porosity with increasing PHBV concentration as well as the polymer itself. Mechanical properties in the 5% PHBV blend showed a 7% increase in compression modulus, but foams showed a 180% improvement in modulus over pure PCL. Compression strength increased 8% in the unfoamed system while the foams increased by 462%. This indicates strengthening of the foam wall whilst retaining CO_2_ miscibility, and ensures both mechanical performance and effective foams. Acoustic performance was relatively invariant across the unfoamed systems as well as the foams, indicating that the polymer more than the foam architecture contributed. Evaluating the foams for low and high frequency response, it can be inferred that while marginal, a greater benefit was obtained at high frequency than at low frequency. Thermal performance scaled with concentration of PHBV, but foams had an accentuating effect on the magnitudes. The range of thermal conductivity across all blends varied by just 20%, but the corresponding foams showed a 80% increase. From the perspective of a biobased foam, manufactured using CO_2_, the thermal performance can be scaled using the thermal diffusivity, which reflects the rate at which heat transfers in a material. As shown in Figure 11, using the relationship (Equation (10)) for thermal diffusivity, *α* (m^2^/sec), in terms of thermal conductivity, *ƛ* (W/(m·K); density, ρ (kg/m^3^); and specific heat, *C_p_* (J/(kg·K), a Styrofoam alternative can be obtained for thermal insulation with improved mechanical performance over pure PCL resulting from adding PHBV.

(10) $$\alpha =\frac{ƛ}{\rho C_{p}}$$

## Figures and Tables

**Table 1:** Table of values for the morphological and performance properties of the unfoamed and foam composites.

Sample (% PHBV)	0	5	15	25	35	45	55	65
**Density unfoamed (g/cm^3^)**	1.099 ± 0.006	1.106 ± 0.003	1.108 ± 0.051	1.157 ± 0.057	1.16 ± 0.049	1.175 ± 0.034	1.188 ± 0.019	1.19 ± 0.009
**Density foam (g/cm^3^)**	0.33 ± 0.002	0.3 ± 0.001	0.364 ± 0.003	0.512 ± 0.003	0.585 ± 0.001	0.687 ± 0.001	0.88 ± 0.002	1.055 ± 0.004
**Expansion ratio**	3.4	3.65	3.26	2.25	2.17	1.74	1.31	1.127
**Percentage porosity (%) (Pycnometer)**	69.06 ± 0.125	70.15 ± 0.079	65 ± 0.097	57 ± 0.313	50.25 ± 0.204	38.79 ± 0.0405	19.81 ± 0.137	10.51 ± 0.002
**Cell Density (cell/µm^3^)**	8.59 × 10^7^	7.75 × 10^7^	1.21 × 10^6^	2.73 × 10^6^	1.95 × 10^6^	3.40 × 10^7^	5.66 × 10^7^	4.63 × 10^7^
**Cell Size (µm)**	134.07 ± 17.07	143.26 ± 16.40	117.23 ± 30.09	81.54 ± 24.59	79.97 ± 20.74	123.48 ± 12.96	78.3 ± 21.05	61.65 ± 12.17
**Void fraction (%)**	71.5	72.8	68.7	60	53.9	42.5	25.9	13
**Cell wall thickness (µm)**	26.21 ± 0.017	24.55 ± 0.008	25.83 ± 0.045	27.66 ± 0.037	33.6 ± 0.010	68.15 ± 0.0064	75.5 ± 0.021	121.69 ± 0.024
**A.T. Conductivity (W/m·K) (unfoamed)**	0.2407 ± 0.034	0.2244 ± 0.017	0.2561 ± 0.021	0.2435 ± 0.007	0.2953 ± 0.005	0.2731 ± 0.002	0.2856 ± 0.0081	0.3007 ± 0.0008
**C_p_ (J/Kg·K) (Unfoamed)**	485.2	510.67	481.08	472.11	420.47	404.6	399.46	473
**Thickness (m) (Unfoamed)**	0.02 ± 0.001	0.02 ± 0.003	0.02 ± 0.001	0.02 ± 0.003	0.02 ± 0.002	0.02 ± 0.001	0.02 ± 0.128	0.02 ± 0.424
**A.T. Conductivity (W/m·K) (Foams)**	0.085 ± 0.0034	0.076 ± 0.0017	0.099 ± 0.0021	0.120 ± 0.0077	0.157 ± 0.0052	0.181 ± 0.0029	0.232 ± 0.0082	0.288 ± 0.0008
**C_p_ (J/Kg·K) (Foams)**	486.33	511.06	483.8	472.76	420.59	407.81	401.74	476.75
**Thickness (Foams)**	0.02 ± 0.007	0.02 ± 0.003	0.02 ± 0.005	0.02 ± 0.003	0.02 ± 0.002	0.02 ± 0.001	0.02 ± 0.001	0.02 ± 0.004
**Thermal Dffusivity (m^2^/s) (Foams)**	5.29 × 10^7^ ± 0.003	4.96 × 10^7^ ± 0.002	5.62 × 10^7^ ± 0.002	4.96 × 10^7^ ± 0.007	6.38 × 10^7^ ± 0.005	6.46 × 10^7^ ± 0.003	6.56 × 10^7^ ± 0.005	5.73 × 10^7^ ± 0.001
**Compression Modulus (MPa) Unfoamed**	7.14 ± 0.425	7.69 ± 0.144	8.33 ± 0.342	9.26 ± 0.244	10 ± 0.420	9 ± 1.160	4.29 ± 0.470	5.56 ± 3.033
**Compression Strength (MPa) Unfoamed**	0.5 ± 0.431	0.54 ± 1.613	0.55 ± 0.13	0.57 ± 0.66	0.6 ± 0.08	0.45 ± 0.03	0.3 ± 0.28	0.5 ± 0.025
**Compression Modulus (MPa) Foams**	0.89 ± 0.008	2.5 ± 0.006	1.86 ± 0.004	7.5 ± 0.005	12.5 ± 0.003	6.67 ± 0.002	5.71 ± 0.005	5.2 ± 0.003
**Compression Strength (MPa) Foams**	0.16 ± 0.047	0.9 ± 0.042	0.5 ± 0.022	1.7 ± 0.012	2.5 ± 0.005	0.6 ± 0.004	0.55 ± 0.05	0.37 ± 0.007
**Sound Absorption (500 Hz) Unfoamed**	0.9046 ± 0.056	0.9135 ± 0.061	0.9120 ± 0.035	0.9015 ± 0.035	0.8930 ± 0.031	0.9022 ± 0.06	0.8915 ± 0.034	0.8852 ± 0.049
**Sound Absorption (4000 Hz) Unfoamed**	0.8740 ± 0.012	0.8962 ± 0.005	0.9085 ± 0.019	0.9092 ± 0.018	0.8987 ± 0.027	0.8965 ± 0.015	0.8971 ± 0.014	0.8977 ± 0.004
**Sound Absorption (500 Hz) Foam Core**	0.9204 ± 0.016	0.9310 ± 0.024	0.9149 ± 0.01	0.9120 ± 0.06	0.9053 ± 0.025	0.9075 ± 0.02	0.9023 ± 0.013	0.8918 ± 0.02
**Sound Absorption (4000 Hz) Foam Core**	0.9113 ± 0.015	0.9159 ± 0.013	0.9191 ± 0.08	0.9264 ± 0.015	0.8977 ± 0.024	0.9155 ± 0.081	0.9019 ± 0.05	0.8997 ± 0.044

**Table 2:** Melting temperature and enthalpy values for both the unfoamed and foamed samples.

Second DSC Cycle		Melting Temp. (°C)		(J/g)	(J/g)	(J/g)	First DSC Cycle		Melting Temp. (°C)		(J/g)	(J/g)	(J/g)
Unfoamed PCL/PHBV	T_m1_	T_m2_	T_m3_	ΔHm1	ΔHm2	ΔHm3	PCL/PHBV Foams	T_mf1_	T_mf2_	T_mf3_	ΔHm1	ΔHm2	ΔHm3
0_BV	58.35	N/A	N/A	46.90	N/A	N/A	0_BV	59.6	NA	NA	10.64	NA	NA
5_BV	58.44	N/A	164.2	71.70	N/A	12.69	5_BV	60.39	NA	167.82	9.25	NA	18.67
15_BV	58.91	158.71	168.2	51.20	15.97	15.97	15_BV	61.76	159.06	168.17	17.96	23.32	26.80
25_BV	58.32	160.64	169.7	58.60	16.91	20.74	25_BV	60.26	161.14	170.65	10.19	25.63	22.52
35_BV	58.78	164.09	171.28	54.83	27.90	12.26	35_BV	61.94	163.45	170.53	6.17	26.45	9.41
45_BV	58.52	N/A	168.1	36.34	N/A	31.64	45_BV	60.84	168.28	173.16	12.75	43.28	10.01
55_BV	59.47	N/A	167.55	54.82	N/A	68.54	55_BV	60.14	167.65	172.52	4.95	31.28	5.34
65_BV	58.78	N/A	168.83	49.67	N/A	60.35	65_BV	61.48	167.99	172.86	10.31	41.43	9.77
75_BV	57.93	166.8	171.38	34.15	28.81	16.56	75_BV	NA	NA	NA	NA	NA	NA
85_BV	57.1	165.46	171.17	31.78	36.25	16.40	85_BV	NA	NA	NA	NA	NA	NA
100_BV	N/A	N/A	174.41	N/A	N/A	82.95	100_BV	NA	NA	NA	NA	NA	NA

**Table 3:** Crystallization temperature and enthalpy values of the solid composites and foams.

First DSC Cycle	T_c1_	T_c2_	H_c1_	H_c2_	First DSC	T_cf1_	T_cf2_	H_cf1_	H_cf2_
					Cycle				
Unf. PCL/PHBV	(°C)	(°C)	(J/g)	(J/g)	PCL/PHBV Foams	(°C)	(°C)	(J/g)	(J/g)
0_BV	33.01	N/A	−2.00	N/A	0_BV	33.3	N/A	−2.87	N/A
5_BV	33.09	73.2	−2.41	−2.61	5_BV	34.77	74.5	−2.10	−2.17
15_BV	32.7	95.59	−1.78	−15.75	15_BVf	32.01	101.18	−4.78	−36.51
25_BV	32.26	101.27	−2.26	−18.72	25_BVf	32.29	101.22	−3.40	−22.37
35_BV	32.57	111.58	−1.76	−14.53	35_BVf	31.9	112.98	−1.48	−14.04
45_BV	36.17	113.75	−1.61	−10.75	45_BVf	35.3	112.92	−5.48	−34.39
55_BV	36.21	113.29	−3.57	−24.91	55_BVf	34	114.04	−2.73	−16.08
65_BV	34.67	113.77	−2.18	−14.81	65_BVf	34.03	113.1	−5.09	−34.75
75_BV	36.33	114.52	−3.61	−21.12	75_BVf	N/A	N/A	N/A	N/A
85_BV	31.9	115.12	−2.29	−18.31	85_BVf	N/A	N/A	N/A	N/A
100_BV	N/A	121.43	N/A	−26.02	100_BVf	N/A	N/A	N/A	N/A

**Table 4:** Comparison between theoretical and experimental measured thermal conductivity values.

Sample Type	Theoretical Values (W/m.K)	Experimental Values (W/m.K)	Difference (%)
0_BV	0.079	0.0853	7.3
5_Bv	0.0691	0.0762	9.3
15_BV	0.0885	0.0988	10.4
25_BV	0.1081	0.1201	10
35_BV	0.1484	0.1571	5.5
45_BV	0.1724	0.1806	4.5
55_BV	0.2314	0.232	0.3
65_BV	0.2848	0.2875	0.9

## Data Availability

Not applicable.

## Nomenclature

		**Units**
A	Sample area	cm^2^
*c*	Particle velocity	m/S
C_p_	Specific heat capacity at constant pressure	J/Kg·K
C_wt_	Cell wall thickness	µm
E_r_	Expansion ratio	
*f*	Frequency	Hz
F	foam	
H°_m_	Heat of Fusion	J/g
H_m_	Enthalpy of Melting	J/g
M	Magnification	µm
N	number of cells	
N_f_	Cell density	cell/µm^3^
N_s_	Cell size	µm
P_foam_	Foaming pressure	MPa
P_sat_	Saturation pressure	MPa
*R*	Thermal resistance value	m·K/W
t	sample thickness	m, mm
T_c_	Crystallization temperature	°C
T_c2_	Second cycle T_c_ (unfoamed polymer)	°C
T_cf1_	First cycle T_c_ (foam)	°C
T_foam_	Foaming temperature	°C
T_m_	Melting temperature	°C
T_m_°	Equilibrium Melting Point	°C
T_m1_	First melting temperature (unfoamed polymer)	°C
T_m2_	Second melting temperature (unfoamed polymer)	°C
T_m3_	Third melting temperature (unfoamed polymer)	°C
T_mf1_	First melting temperature (foams)	°C
T_mf2_	Second melting temperature (foams)	°C
T_mf3_	Third melting temperature (foams)	°C
T_sat_	Saturation temperature	°C
V_f_	Void fraction	%
X_BV	Percentage weight concentration of PHBV (unfoamed)	%
X_BVf	Percentage weight concentration of PHBV (foam)	%
**Greek Symbols**		
Δ(.)	Increment of a quantity	
ϴ	Volume fraction	%
λ	Individual acoustic wavelength	m
ƛ	Thermal Conductivity	W/m·K
ρ	Density	g/cm^3^
ϕ	Weight percentage of dispersed phase	%
ꭓ	Percentage crystallinity	%
**Subscript**		
cv	convection	
f	foam	
g	gas	
p	polymer	
r	radiation	
s	size	
sat	saturation	
sol	solid phase	
1, 2	indices for cycles	
